# Evaluation of Adverse Reactions Induced by Anti-Tuberculosis Drugs in Hospital Pulau Pinang

**DOI:** 10.21315/mjms2018.25.5.10

**Published:** 2018-10-30

**Authors:** Cheah Meng Fei, Hadzliana Zainal, Irfhan Ali Hyder Ali

**Affiliations:** 1 Department of Pharmacy, Hospital Tawau, PO Box 67, 91007 Tawau, Sabah, Malaysia; 2 School of Pharmaceutical Sciences, Universiti Sains Malaysia, 11800 Universiti Sains Malaysia, Pulau Pinang, Malaysia; 3 Respiratory Department, Hospital Pulau Pinang, Jalan Residensi, 10990 Georgetown, Pulau Pinang, Malaysia

**Keywords:** adverse drug reactions, anti-tuberculosis drugs, incidence, relationship, treatment outcomes

## Abstract

**Background:**

The use of multi-drug regimens in tuberculosis (TB) treatment has been associated with undesirable adverse drug reactions (ADRs). This study aims to assess the incidence and impact of ADRs on TB treatment in Hospital Pulau Pinang.

**Methods:**

This cross-sectional study was conducted via retrospective review of outpatients’ medical records. Details regarding ADRs were identified by a pharmacist and verified by a consultant respiratory physician.

**Results:**

A total of 91 cases, out of 210 patients enrolled in this study, were detected with 75 patients (35.7%) experienced at least one ADR. The three most common ADRs detected were cutaneous adverse drug reactions (CADRs) (21.0%), drug-induced hepatitis (DIH) (7.1%) and gastrointestinal disturbance (4.8%). Pyrazinamide was the most common causative agent and 15.7% of all TB patients required treatment modification due to ADRs. Females were shown to have a higher tendency to develop ADRs than the males in this study (*P* = 0.009). The development of ADRs was shown not to affect the TB treatment outcomes (*P* = 0.955).

**Conclusion:**

The incidence of ADRs in this study was high so it is important to identify the risk factors for ADRs and the individuals who have those risk factors when initiating anti-TB drugs. These individuals require special attention when anti-TB drugs are initiated.

## Introduction

Tuberculosis (TB) is an infectious disease caused by *Mycobacterium tuberculosis*. It mainly affects the lungs (pulmonary tuberculosis [PTB]) but it can also affect other sites of the body (extrapulmonary tuberculosis [EPTB]) (1). TB remains as a major health problem globally and is a leading cause of death worldwide. In 2015, there were an estimated 10.4 million of incident cases and an estimated 1.4 million deaths from TB worldwide (1). In Malaysia, the number of TB cases continues to rise and these cases lead to high rates of morbidity and mortality (2). The number of new TB cases in Malaysia increased from 15,000 in 2005 to 19,251 in 2011 (2). The latest estimates showed that there were 27,000 new TB cases in Malaysia in 2015 (1).

The aims of TB treatment are to cure the patient and to restore the quality and productivity of life, to prevent death from active TB or its late effects, to prevent relapse, to reduce transmission and to prevent the development and transmission of drug resistance (3). The currently recommended treatment for new cases of drug-susceptible TB is a regimen of four first-line drugs: isoniazid (H), rifampicin (R), ethambutol (E) and pyrazinamide (Z) (1). In 1995, the World Health Organization (WHO) launched the Directly Observed Treatment Short course (DOTS) (2). DOTS is a standard regimen which requires the TB patient to continually take weight-based drug combinations of H, R, E, Z and/or streptomycin (S) for a designated time period and it is currently practised in Malaysia (2, 3). In Malaysia, the anti-TB drugs are either supplied as separate-drug regimens or as fixeddose combinations (FDC) (2). Two FDCs were available in Hospital Pulau Pinang in 2015, namely Akurit-4 (E 275 mg, H 75 mg, R 150 mg, Z 400 mg) and Akurit-2 (H 75 mg, R 150 mg). Besides drug administration, a TB booklet is supplied to all patients who are started on anti-TB drugs for the recording and monitoring of drug adherence purposes.

According to WHO (4), an adverse drug reaction (ADR) is defined as “any response to a drug which is noxious and unintended, and which occurs at doses normally used in man for prophylaxis, diagnosis, or therapy of disease, or for the modification of physiological function.” ADRs cause serious problems like morbidity, mortality and high cost of patient care (5). Based on a systematic review conducted by Singh et al., the overall prevalence of ADRs with first-line anti-TB drugs varied from 8.4% to 83.5% (6). The use of multi-drug regimens in TB treatment has been associated with undesirable ADRs at varying degrees of severity, such as hepatotoxicity, skin rashes, gastrointestinal disturbances, neurological disorders and musculoskeletal disorders (6–24). ADRs were observed more commonly in the intensive phase of TB treatment and did not differ between intermittent or daily intake of anti-TB drugs (6). The occurrence of ADRs was influenced by multiple factors (6). The management of ADRs due to anti-TB drugs depends on the severity of the reactions. It ranges from regular monitoring, symptomatic therapy, hospitalisation to modification of the anti-TB regimen (3). Based on Lv et al., when compared with patients without ADRs, patients who developed ADRs were more likely to have positive smear test results at the end of the intensive phase and unsuccessful TB treatment outcomes (20).

Through a retrospective study conducted in Hospital Pulau Pinang, Malaysia by Kurniawati et al. in 2012, it was reported that 15.8% of the patients experienced ADRs (7). The most common ADR was skin reaction (7.8%), followed by hepatotoxicity (2.6%) and gastrointestinal reactions (2.5%) (7). The management of ADRs was mostly included adding on medication (8.6%), withholding treatment regimens (6.1%), continuing medication without any changes (0.9%) and only 0.2% of the patients needed alteration of the anti-TB regimen (7). Factors such as alcohol consumption, drug abuse and maintenance phase were found to be significantly associated with the occurrence of ADRs (7). Although conducted in the same hospital, the study did not address the impact of ADRs on TB treatment in the majority of the studies. Hence, this study aims to obtain an overview of ADRs due to anti-TB drugs and to evaluate the impact of such ADRs on TB treatment among patients in Hospital Pulau Pinang.

## Methods

### Study Design and Population

This cross-sectional study was conducted from 9 January 2017 to 19 March 2017 in Chest Clinic, Hospital Pulau Pinang, via retrospective review of outpatients’ medical records. The population consisted of patients who were newly diagnosed with tuberculosis and were treated with anti-TB drugs in the year 2015.

### Sample Selection

Included in this study were: i) patients who were newly diagnosed to have PTB from 1 January 2015 to 31 December 2015; ii) patients who were taking first-line anti-TB drugs, namely H, R, E, Z and/or S; and iii) patients aged 18 years old and above. Patients were excluded from the study for the following reasons: i) incomplete medical records; ii) presented with signs and symptoms of underlying diseases or conditions that overlapped with those of ADRs.

The estimated minimum sample size required for this study was 204. This figure was arrived at by assuming that a 95% chance of our estimates being within ± 5% of the true proportion, assuming that 15.8% of the patients developed ADRs after taking anti-TB drugs based on Kurniawati et al. (7)

The medical records of all eligible patients were reviewed. Patients were followed up until case closure based on the treatment outcome “cure”, “treatment completed”, “treatment failure”, “died” or “default” (3).

### Data Collection and Study Variables

From the medical records, the relevant data needed were recorded in a data collection form. Data that was extracted included the patients’ demographic and clinical characteristics, ADRs due to anti-TB drugs and TB treatment outcomes. The TB treatment outcomes for all patients were categorised into successful outcomes (defined as “cure” or “treatment completed”) and unsuccessful outcomes (defined as “treatment failure”, “died” or “default”). ADRs in this study were identified from the medical records, based on the definition as described below, by Investigator 1 (a pharmacist) and verified by Investigator 2 (a consultant respiratory physician).

1. Drug-induced hepatitis (DIH)
  DIH was defined as an increase in serum transaminase level that was more than three times of the upper limit of normal (ULN) for patients with symptoms suggestive of hepatitis or five times of the ULN for those without symptoms (2). DIH was also considered when there was an increase in the total bilirubin count that was more than two times of the ULN (25).
2. Anemia
  Anemia was defined as when the hemoglobin concentration is less than 11 g/dL in male or less than 10 g/dL in female for patients without a history of anemia or more than 1 g/dL drop in hemoglobin concentration after the initiation of anti-TB drugs (20).
3. Thrombocytopenia
  Thrombocytopenia was defined as a drop in platelet count equal to or less than 150 × 10^9^/L (20).
4. Hypokalemia
  Hypokalemia was defined as when the serum potassium level is less than 3.5 mmol/L.
5. Others
  Other ADRs such as cutaneous adverse drug reactions (CADRs), gastrointestinal disturbances, visual disturbances, peripheral neuropathy, joint pain and leg swelling were determined based on symptoms.

### Data Management and Statistical Analysis

Demographic information and clinical characteristic of patients, incidence of ADRs, types of ADRs, onset of ADRs, management and outcomes of ADRs and TB treatment outcomes were descriptively reported either in percentage or median (interquartile range) [IQR]. The Chi-squared or Fisher’s exact test was used to assess the factors associated with the development of ADRs among patients and to assess the TB treatment outcomes in patients who developed ADRs. For all statistical tests performed, the significance level was set at *P* < 0.05.

## Ethics of Study

This study was approved by Medical Research and Ethics Committee (MREC), Ministry of Health (MOH), Malaysia [(6)KKM/NIHSEC/P16-1605] via National Medical Research Registry (NMRR) with the registration number NMRR-16-1972-32785. All data obtained from the medical records was kept confidential.

## Results

### Demographic and Clinical Characteristics of Patients

A total of 581 patients were newly diagnosed to have tuberculosis. Of those patients, 437 were diagnosed to have PTB. After further excluding patients based on the exclusion criteria (96 incomplete medical records, 38 relapse cases, 10 had signs and symptoms of underlying diseases or conditions that overlapped with those of ADRs, eight were not in the first-line regimen, 15 aged less than 18 years old, seven changed diagnosis during treatment and 53 medical records were not found), only 210 were enrolled in this study.

Table 1 shows the demographic and clinical characteristics of the 210 patients. The median (IQR) age of the patients was 52.00 years (35.75, 60.00 years). All the recruited patients were Malaysian and 99.0% of them stayed in urban area. The median (IQR) body weight of the patients was 50.00 kg (44.25, 59.00 kg). The most common types of comorbidities that the patients had were diabetes mellitus (26.2%), hypertension (14.7%), dyslipidemia (9.5%), ischemic heart disease (4.8%), chronic kidney disease (4.8%) and cerebrovascular accident (2.9%).

### Anti-TB Regimen and Treatment Outcomes

Most of the patients (87.6%) underwent both the intensive and maintenance treatment phases. A majority of the duration of the intensive phase was 2 months (minimum of 3 days, maximum of 9 months) while the duration of the maintenance phase was 4 months (minimum of 10 days, maximum of 10 months and 2 weeks). It was noticed that 80.0% of the patients were given Akurit-4 in the intensive phase. The remaining patients were given modified regimens such as EHR and HRZ due to underlying clinical conditions. For the maintenance phase, most of the patients were given Akurit-2 (41.0%) or HR (44.3%). One patient was given HE for 9 months for both the intensive and maintenance treatment phases.

Out of the 210 patients, 176 (83.8%) achieved successful outcomes, 18 (8.6%) died, 15 (7.1%) defaulted treatment and one (0.5%) experienced treatment failure.

### ADRs due to Anti-TB Drugs

A total of 75 patients experienced at least one ADR (35.7%), including 61 patients with one ADR, 12 patients with two ADRs and two patients with three ADRs. Hence, a total of 91 cases were detected. The types and incidences of ADRs detected are shown in Table 2. The most common ADR encountered was CADR (21.0%). The symptoms of CADR were rashes and itchiness on the skin. DIH (7.1%) and gastrointestinal disturbance (4.8%) were also common among patients. Rare ADRs which were classified under “Others” included thrombocytopenia (one case), anemia (two cases), hypokalemia (one case) and leg swelling (one case).

Out of the 91 ADRs, 81 (89.0%) occurred in the intensive treatment phase. The median (IQR) onset of the ADRs was 15 days (12, 38 days), with the minimum onset of a few hours and the maximum onset of 330 days. However, the onset of four cases was unknown. The details of this section can be found in Appendices 1 and 2.

The suspected anti-TB drugs for most of the ADR cases (70.3%) were unknown (Table 2). Two CADR cases were suspected to be caused by a combination of two drugs, namely EZ and RZ, respectively.

In terms of the management of the ADRs, 48 cases (52.7%) required symptomatic therapy, 25 (27.5%) required examination and nine (9.9%) required hospitalisation (Table 3). There were 33 ADR cases (36.3%) that required anti-TB regimen modification (Table 4). The two common forms of anti-TB regimen modification were interruption and discontinuation. Interruption was most common (53.3%) in DIH and discontinuation was most common (83.3%) in visual disturbance.

Most of the patients who developed ADRs either recovered fully (49.5%) or were recovering from the reactions (11.0%) (Appendix 3). It was noticed that one patient (1.1%) died of DIH. The outcome of 34 ADR cases (37.4%) was unknown.

### Relationship between ADRs and Patients’ Characteristics

The proportion of patients, who developed ADRs based on their demographic and clinical characteristics and the relationship, is shown in Table 5. Of all the factors, only sex was found to have a significant relationship with the development of ADRs (*P* = 0.009). Female patients had a higher tendency (50.0%) to develop ADRs than male patients (30.5%).

### TB Treatment Outcomes in Patients Who Developed ADRs

Table 6 shows patients’ characteristics and the impact of ADRs on TB treatment outcomes. Although the development of ADRs did not affect the TB treatment outcomes (*P* = 0.955), smoking (*P* = 0.031) and the presence of comorbidities (*P* = 0.035) significantly affected the treatment outcomes.

## Discussion

Among 210 patients included in this study, 75 of them (35.7%) experienced at least one ADR, with a total of 91 cases detected. CADRs were most commonly detected (21.0%). The other frequent ADRs (in descending order) were DIH, gastrointestinal disturbance, peripheral neuropathy, visual disturbance and joint pain. Although studies were conducted in the same hospital, this study detected higher incidences of overall ADRs and CADRs compared to Kurniawati et al. (7) and Tan et al. (14), respectively. However, the ADR incidence and its order could not be compared to most of the similar studies (7, 9, 17, 19, 20) due to differences in the population sampled, study setting, study duration and methodology used to detect and classify the ADRs.

In this study, most of the patients who developed CADRs had mild itchiness. Only 7.5% of the patients developed skin rashes after taking anti-TB drugs. However, the type of rash was not mentioned in the medical records. In fact, CADRs, especially maculopapular rash, had been shown to occur very commonly among patients who were taking anti-TB drugs (7, 11, 14, 24). The next common ADR detected among patients in this study was DIH. Most of the patients in this study developed asymptomatic rise in serum transaminase level. Only one patient developed hyperbilirubinemia after taking anti-TB drugs. As shown in previous literatures, the incidence of drug-induced hepatitis (DIH) ranged from 2.0% to 39.0% worldwide (6, 25). The symptoms of gastrointestinal disturbance experienced by patients in this study included nausea and vomiting, abdominal bloating, loss of appetite and gastritis. In fact, some studies showed that gastrointestinal disturbance was the most common ADR detected with the intake of anti-TB drugs in their setting (10, 19, 23, 24). Another ADR experienced by patients in this study was joint pain. This might probably be caused by pyrazinamide- or ethambutol-induced hyperuricemia (6). Other rare ADRs detected in this study such as hematological disorders and leg swelling had been discussed in previous studies and case reports (6, 8, 9, 15, 26–32).

Most of the ADRs in this study occurred in the intensive treatment phase. This finding was similar to the one in previous studies (8, 9, 14, 17, 18, 20) where most of the ADRs occurred within the first 2 months after anti-TB drugs were initiated. Knowing the onset of ADRs is helpful in early detection and prompt management of ADRs. Hence, it is essential for healthcare professionals to counsel patients for early identification of ADRs especially in the intensive treatment phase. Patients should be advised to consult healthcare professionals should the above-mentioned signs and symptoms of ADRs occur. Besides, regular monitoring of patients during the intensive treatment phase is essential for early detection of ADRs.

This study showed that among those ADRs for which suspected drugs were known, Z was the most common drug causing ADRs. According to literature, all first-line anti-TB drugs had been associated with the development of CADRs, with Z being the most common offending drug, followed by S, E, R and H (2, 3, 6, 14). Literature reviewed also showed that DIH was usually caused by H, R and Z, with Z being the most hepatotoxic and R being the least hepatotoxic (2, 3). In this study, Z was found to be the suspected drug for all DIH cases, except those cases for which suspected drugs were unknown.

In general, the management of ADRs due to anti-TB drugs in this study was similar to that mentioned in the literature (2, 3, 6, 14) except for the management of peripheral neuropathy. In this study, most of the patients who developed peripheral neuropathy had their dose of pyridoxine increased from the prophylactic dose of 10 mg daily to 20–30 mg daily. Such management was different from the WHO guidelines where patients who developed peripheral neuropathy should be prescribed with 50 to 70 mg daily of pyridoxine (3). This study also showed that 15.7% of all TB patients required treatment modification due to ADRs. A prospective study by Lv et al. found that only 7.6% of TB patients required modifying their TB treatment due to ADRs (20).

Of all the patients’ demographic and clinical characteristics, only sex was found to have a statistically significant relationship with the development of ADRs. Female patients had a higher tendency to develop ADRs due to anti-TB drugs than male patients in this study. There were many studies which showed similar findings (8, 9, 13, 16, 21). The possible mechanisms behind such findings included hormonal fluctuations at different stages of life, such as pregnancy and menarche, which modify drug responses (9, 21). The interactions between anti-TB drugs and contraceptive medications might also favour the occurrence of ADRs (9, 21). Such findings suggest the need for special precautions, medication counselling and more intensive monitoring when anti-TB drugs are prescribed to female patients.

This study showed that the development of ADRs did not affect the TB treatment outcomes of the patients. There were only a few studies which described the impact of ADRs on TB treatment outcomes. Shin et al. showed that ADRs occurred frequently among MDR-TB patients but did not negatively impact treatment outcomes (33). However, Lv et al. showed that patients who developed ADRs were more likely to have unsuccessful TB treatment outcomes (20). Besides, based on a study conducted by Shang et al., compared with those without anti-TB-induced liver injury (ATLI), ATLI patients had a 9.3-fold risk of unsuccessful TB treatment outcomes (25). TB patients with unsuccessful outcomes had a higher risk to develop MDR-TB and consequently had a lower probability to be cured (34).

This study had a few limitations. It was a retrospective study that reviewed handwritten medical records and hence, missing data was inevitable. The use of electronic medical records in the future might help solve this problem. The duration of treatment could not be determined properly because the patients’ TB booklets were not available and no documentation was made regarding that for some patients. We were also unable to determine the onset and outcomes of the ADRs accurately because some ADRs, especially those that required laboratory investigations, could only be performed during the patients’ follow up. Those ADRs might have occurred and the outcomes of the ADRs could have been seen before the date of the follow up. Nevertheless, this study serves to give an insight of the possible ADRs that might be experienced by patients who are taking anti-TB drugs.

The difference in study design and setting among different studies disallowed us to compare the results obtained. For example, the first-line anti-TB regimen used in different countries might be different. Besides, some studies were conducted in patients who were hospitalised where the monitoring of patients was more intensive and hence increased the chance for ADR detection. Therefore, the results obtained could not be generalised to the whole TB population in Hospital Pulau Pinang. Future studies should be conducted by prospective review of all TB patients in the hospital.

Lastly, according to literature review, different risk factors were associated with different ADRs. Perhaps future studies could be conducted by focusing on one particular ADR and its contributing factors. Besides, it was noticed that patients’ serum uric acid levels were not measured in this study. Since joint pain was thought to be caused by ethambutol- or pyrazinamide-induced hyperuricemia, patients who are prescribed these two drugs should have their serum uric acid levels measured in the future.

## Conclusion

This study showed that 35.7% of patients presented at least one ADR, with a total of 91 cases detected. The most common ADR detected was CADR. Most of the ADRs occurred in the intensive treatment phase. Among those ADRs for which the suspected drugs were known, Z was the most common causative agent. Most of the ADRs could be managed well by giving symptomatic therapy. However, 15.7% of all TB patients required treatment modification due to ADRs. Most of the patients who developed ADRs either recovered fully or were recovering from the reactions. Females were shown to have a higher tendency to develop ADRs than the males in this study. However, the development of ADRs was shown not to affect the TB treatment outcomes.

Although most ADRs in this study could be managed well, the incidence was high. First-line anti-TB drugs are administered as a combination of drugs. Hence, it is difficult to evaluate the ADRs of the individual component. A thorough knowledge of pharmacokinetics and possible ADRs of the drugs, as well as the interactions among those drugs, allows healthcare professionals to treat TB patients more safely and effectively. Besides, it is also important to identify the risk factors for ADRs and individuals who have those risk factors when initiating anti-TB drugs, especially those who are initiated on Z. These individuals require special precautions, more intensive monitoring and medication counselling when anti-TB drugs are initiated. Lastly, this study highlights the importance of developing systems and strategies for proper monitoring and amelioration of ADRs due to anti-TB drugs in order to improve the quality of patient care.

## Figures and Tables

**Table 1:** Patients’ demographic data and clinical characteristics (*N* = 210)

Variable	*N* (%)
Age (years)	
< 50	93 (44.3)
≥ 50	117 (55.7)
Sex	
Male	154 (73.3)
Female	56 (26.7)
Race	
Malay	66 (31.4)
Chinese	112 (53.3)
Indian	26 (12.4)
Others	6 (2.9)
Location	
Urban	208 (99.0)
Rural	2 (1.0)
Smoking	
Yes	93 (44.3)
No	114 (54.3)
No data	3 (1.4)
Alcohol use	
Yes	21 (10.0)
No	186 (88.6)
No data	3 (1.4)
Substance abuse	
Yes	19 (9.0)
No	188 (89.5)
No data	3 (1.4)
HIV	
Yes	6 (2.9)
No	139 (66.2)
No data	65 (31.0)
Comorbidity	
Yes	120 (57.1)
No	90 (42.9)
Concurrent medication use	
Yes	107 (51.0)
No	103 (49.0)

**Table 2:** Types of ADR detected, incidence and suspected anti-TB drugs that caused ADRs (*N* = 91)

Type	*N* (%)							Total, *N* (%)	Incidence (%)^a^

	H	R	E	Z	EZ	RZ	Unknown		
CADRs	0 (0.0)	2 (4.5)	1 (2.3)	2 (4.5)	1 (2.3)	1 (2.3)	37 (84.1)	44 (48.4)	21.0
DIH	0 (0.0)	0 (0.0)	0 (0.0)	7 (46.7)	0 (0.0)	0 (0.0)	8 (53.3)	15 (16.5)	7.1
Gastrointestinal disturbance	0 (0.0)	0 (0.0)	0 (0.0)	0 (0.0)	0 (0.0)	0 (0.0)	10 (100.0)	10 (11.0)	4.8
Visual disturbance	0 (0.0)	0 (0.0)	6 (100.0)	0 (0.0)	0 (0.0)	0 (0.0)	0 (0.0)	6 (6.6)	2.9
Peripheral neuropathy	7 (100.0)	0 (0.0)	0 (0.0)	0 (0.0)	0 (0.0)	0 (0.0)	0 (0.0)	7 (7.7)	3.3
Joint pain	0 (0.0)	0 (0.0)	0 (0.0)	0 (0.0)	0 (0.0)	0 (0.0)	4 (100.0)	4 (4.4)	1.9
Others	0 (0.0)	0 (0.0)	0 (0.0)	0 (0.0)	0 (0.0)	0 (0.0)	5 (100.0)	5 (5.5)	2.4
Total	7 (7.7)	2 (2.2)	7 (7.7)	9 (9.9)	1 (1.1)	1 (1.1)	64 (70.3)	91 (100.0)	–

a The denominator was 210.

**Table 3:** Management of ADRs (*N* = 91)

Type	*N*	*N* (%)		

		Symptomatic therapy^a^	Examination^b^	Hospitalisation
CADRs	44	31 (70.5)	9 (20.5)	2 (4.5)
DIH	15	1 (6.7)	3 (20.0)	6 (40.0)
Gastrointestinal disturbance	10	6 (60.0)	4 (40.0)	0 (0.0)
Visual disturbance	6	0 (0.0)	4 (66.7)	0 (0.0)
Peripheral neuropathy	7	5 (71.4)	1 (14.3)	1 (14.3)
Joint pain	4	2 (50.0)	2 (50.0)	0 (0.0)
Others	5	3 (60.0)	2 (40.0)	0 (0.0)
Total	91	48 (52.7)	25 (27.5)	9 (9.9)

a Symptomatic therapy for ADRs such as liver protective drugs, drugs to alleviate skin rashes and gastrointestinal disturbances, but not including anti-TB regimen modification.

b Physical examination or monitoring only and no drugs prescribed.

**Table 4:** Anti-TB regimen modification due to ADRs (*N* = 91)

Type	*N*	*N* (%)		

		Anti-TB regimen modification	Forms of anti-TB treatment regimen modification	

			Interruption	Discontinuation
CADRs	44	15 (34.1)	13 (29.5)	8 (18.2)
DIH	15	10 (66.7)	8 (53.3)	7 (46.7)
Gastrointestinal disturbance	10	2 (20.0)	2 (20.0)	0 (0.0)
Visual disturbance	6	6 (100.0)	1 (16.7)	5 (83.3)
Peripheral neuropathy	7	0 (0.0)	0 (0.0)	0 (0.0)
Joint pain	4	0 (0.0)	0 (0.0)	0 (0.0)
Others	5	0 (0.0)	0 (0.0)	0 (0.0)
Total	91	33 (36.3)	24 (26.4)	20 (22.0)

**Table 5:** Factors associated with the development of ADRs due to anti-TB drugs (*N* = 210)

Variable	*N* (%)		*P*-value

	Without ADRs	With ADRs	
Age (years)			0.605^c^
< 50	58 (62.4)	35 (37.6)	
≥ 50	77 (65.8)	40 (34.2)	
Sex			0.009^c^
Male	107 (69.5)	47 (30.5)	
Female	28 (50.0)	28 (50.0)	
Race			0.738^d^
Malay	40 (60.6)	26 (39.4)	
Chinese	72 (64.3)	40 (35.7)	
Indian	19 (73.1)	7 (26.9)	
Others	4 (66.7)	2 (33.3)	
Location			0.672^c^
Urban	134 (64.4)	74 (35.6)	
Rural	1 (50.0)	1 (50.0)	
Smoking^a^			0.052^c^
No	66 (57.9)	48 (42.1)	
Yes	66 (71.0)	27 (29.0)	
Alcohol use^a^			0.441^c^
No	117 (62.9)	69 (37.1)	
Yes	15 (71.4)	6 (28.6)	
Substance abuse^a^			0.658^c^
No	119 (63.3)	69 (36.7)	
Yes	13 (68.4)	6 (31.6)	
HIV^b^			0.674^d^
No	87 (62.6)	52 (37.4)	
Yes	3 (50.0)	3 (50.0)	
Comorbidity			0.967^c^
No	58 (64.4)	32 (35.6)	
Yes	77 (64.2)	43 (35.8)	
Concurrent medication use			0.607^c^
No	68 (66.0)	35 (34.0)	
Yes	67 (62.6)	40 (37.4)	

a *N* = 207

b *N* = 145

c Chi-squared test

d Fisher’s exact test

**Table 6:** Factors associated with TB treatment outcomes (*N* = 210)

Variable	Outcomes, *N* (%)		*P*-value

	Unsuccessful	Successful	
Age (years)			0.722^c^
< 50	16 (17.2)	77 (82.8)	
≥ 50	18 (15.4)	99 (84.6)	
Sex			0.194^c^
Male	28 (18.2)	126 (81.8)	
Female	6 (10.7)	50 (89.3)	
Race			0.906^d^
Malay	12 (18.2)	54 (81.8)	
Chinese	18 (16.1)	94 (83.9)	
Indian	3 (11.5)	23 (88.5)	
Others	1 (16.7)	5 (83.3)	
Location			>0.95^d^
Urban	34 (16.3)	174 (83.7)	
Rural	0 (0.0)	2 (100.0)	
Smoking^a^			0.031^c^
No	13 (11.4)	101 (88.6)	
Yes	21 (22.6)	72 (77.4)	
Alcohol use^a^			0.352^d^
No	29 (15.6)	157 (84.4)	
Yes	5 (23.8)	16 (76.2)	
Substance abuse^a^			0.096^d^
No	28 (14.9)	160 (85.1)	
Yes	6 (31.6)	13 (68.4)	
HIV^b^			0.533^d^
No	16 (11.5)	123 (88.5)	
Yes	1 (16.7)	5 (83.3)	
Comorbidity			0.035^c^
No	9 (10.0)	81 (90.0)	
Yes	25 (20.8)	95 (79.2)	
Concurrent medication use			0.080^c^
No	12 (11.7)	91 (88.3)	
Yes	22 (20.6)	85 (79.4)	
Development of ADRs			0.955^c^
No	22 (16.3)	113 (83.7)	
Yes	12 (16.0)	63 (84.0)	

a *N* = 207

b *N* = 145

c Chi-squared test

d Fisher’s exact test

## Appendix 1 ADRs and treatment phase encountered (*N* = 91)

**Table:**

Type	Treatment phase *N* (%)	

	Intensive	Maintenance
CADRs	41 (93.2)	3 (6.8)
DIH	15 (100.0)	0 (0.0)
Gastrointestinal disturbance	10 (100.0)	0 (0.0)
Visual disturbance	4 (66.7)	2 (33.3)
Peripheral neuropathy	4 (57.1)	3 (42.9)
Joint pain	3 (75.0)	1 (25.0)
Others	4 (80.0)	1 (20.0)
Total	81 (89.0)	10 (11.0)

## Appendix 2 Onset of ADRs (*N* = 87)

**Table:**

Type	*N*^a^	Onset (day)		

		Median (IQR)^b^	Minimum	Maximum
CADRs	43	14 (11, 23)	A few hours	120
DIH	15	14 (12, 19)	9	55
Gastrointestinal disturbance	7	14 (3, 16)	2	40
Visual disturbance	6	42 (11, 197)	3	330
Peripheral neuropathy	7	61 (21, 131)	4	180
Joint pain	4	52 (41, 104)	38	120
Others	5	20 (11, 84)	10	130
Total	87	15 (12, 38)	A few hours	330

Note:

a The onset of four cases which occurred in the intensive phase was unknown.

b Onset less than 1 day (e.g. a few hours) was considered as 1 day.

## Appendix 3 Outcomes of ADRs (*N* = 91)

**Table:**

Type	*N* (%)				

	Recovered fully	Recovering	Not recovering	Unknown	Fatal
CADRs	23 (52.3)	6 (13.6)	0 (0.0)	15 (34.1)	0 (0.0)
DIH	12 (80.0)	1 (6.7)	0 (0.0)	1 (6.7)	1 (6.7)
Gastrointestinal disturbance	4 (40.0)	0 (0.0)	0 (0.0)	6 (60.0)	0 (0.0)
Visual disturbance	2 (33.3)	1 (16.7)	1 (16.7)	2 (33.3)	0 (0.0)
Peripheral neuropathy	2 (28.6)	0 (0.0)	0 (0.0)	5 (71.4)	0 (0.0)
Joint pain	1 (25.0)	0 (0.0)	0 (0.0)	3 (75.0)	0 (0.0)
Others	1 (20.0)	2 (40.0)	0 (0.0)	2 (40.0)	0 (0.0)
Total	45 (49.5)	10 (11.0)	1 (1.1)	34 (37.4)	1 (1.1)
